# Case of Twins, Accompanied with Embarrassing Circumstances

**Published:** 1818-07-01

**Authors:** M. Leveque


To the above interesting case of Mr. Sheppard's, we shall add a somewhat analogous one from a recent French Journal.

### VII.

### *Case of Twins, accompanied with embarrassing Circumstances.*

By M. Leveque, M. D.

Madam R. 19 years of age, of small stature, and extremely delicate organization, became pregnant shortly after marriage. During the first three months she was much harrassed with giddiness and continual vomiting, which reduced her to such a state of emaciation and debility, that she was forced to use ass's milk for a month, which so far invigorated her constitution that she undertook, and bore well, a journey to Auvergne, the residence of her husband. She returned to Orleans in the seventh month of her pregnancy, when the abdomen was found to have attained a remarkable size, and the motion of the child was scarcely perceptible. At this period Madam R. began to experience the most painful dragging sensations in the lumbar region, which continued till her accouchement, and reduced her to such a feeble and debilitated condition, that it was not at all expected that she would be able to go through the approaching trial of parturition. It is impossible for language to convey an idea of this lady's sufferings during the eight days preceding her confinement. The belly was extended to an enormous size, covered with vesicles; of excessive sensibility, and threatening every moment to burst! The pulse was small and frequent; the vagina and labia pudenda so swelled and tender, that examination with the finger was extremely difficult and painful. The agitation was great; and tho' naturally courageous, the patient became overwhelmed with her sufferings, and uttered the most piercing cries. The pains now became periodical, returning every quarter of an hour, which gave hopes of approaching delivery; but the os uteri remained closed and resisting. Various means were tried to soothe the symptoms, as lavements, fomentations, anodynes, and even the warm bath, but without the least effect. It was now nine o'clock in the evening, and the pains were so excruciating, and the prospect of the approaching night so dreadful, that notwithstanding the profound state of debility and exhaustion in which this unfortunate lady was precipitated, I determined, as a last resource, to try venesection. I opened a vein in the arm, and abstracted a small bason full of blood, which she bore well. The pains vanished, a calm ensued, and she fell into a refreshing sleep

At five in the morning she was awoke by fresh pains; but now the dilatation of the os uteri offered a prospect of delivery. The pains continued till noon, with much irregularity, being frequently suspended by syncope and other evidences of extreme debility. During the two next hours, the head, which presented, made no progress; the pains died away, the pulse sunk, and there was every reason to believe that death was at hand, unless Art came in to the assistance of Nature.

High up as was the head, I managed to apply the forceps; but the great difficulty was the extraction of the fœtus, in consequence of the excessive shortness of the funis. It required the greatest precaution not to separate the cord at the umbilicus; and the ligature was obliged to be applied with the infant's abdomen close to the os externum of the mother. The funis instantly disappeared by retraction, yet there was no hæmorrhage. The placenta came away, together with an extraordinary mass of waters, and the funis was found to measure only 5 inches in length, besides two inches which had been torn off in delivery. The head of a second child was felt by the finger; and by turning and bringing down the feet I might have terminated the affair. But the extreme state of exhaustion in which the lady now was, and the dread of even a slight hæmorrhage, which, under existing circumstances, must have been productive of the most dangerous consequences, induced me to trust to Nature the delivery of the second child. In half an hour the os uteri was completely closed, and Madam R. continued tranquil and easy till the end of the 4th day, when labour pains came on, and continued eighteen hours before proper dilatation took place. The feet presented, and I expedited the delivery. Both children were full grown; and the quantity of liquor amnii was prodigious. A smart milk fever succeeded, but the lady perfectly recovered from this perilous situation, and is now alive and well.

We conceive that the above case conveys an important lesson relative to the tranquilising effects of venesection, in states of *debility, irritability, and pain,* when opium, and anti-spasmodics are too often exhibited with little success.—*Ed.*

